# Tracking global trends in the effectiveness of antibiotic therapy using the Drug Resistance Index

**DOI:** 10.1136/bmjgh-2018-001315

**Published:** 2019-04-11

**Authors:** Eili Y Klein, Katie K Tseng, Suraj Pant, Ramanan Laxminarayan

**Affiliations:** 1 Department of Emergency Medicine, Johns Hopkins School of Medicine, Baltimore, Maryland, USA; 2 Department of Epidemiology, Johns Hopkins Bloomberg School of Public Health, Baltimore, Maryland, USA; 3 Center for Disease Dynamics, Economics & Policy, Washington, District of Columbia, USA

**Keywords:** Antibiotic resistance, public health, low- and middle-income countries, antibiotic consumption, Onehealth

## Abstract

**Background:**

Evaluating trends in antibiotic resistance and communicating the results to a broad audience are important for dealing with this global threat. The Drug Resistance Index (DRI), which combines use and resistance into a single measure, was developed as an easy-to-understand measure of the effectiveness of antibiotic therapy. We demonstrate its utility in communicating differences in the effectiveness of antibiotic therapy across countries.

**Methods:**

We calculated the DRI for countries with data on antibiotic use and resistance for the disease-causing organisms considered by the WHO as priority pathogens: *Acinetobacter baumannii*, *Escherichia coli*, *Klebsiella pneumoniae*, *Pseudomonas aeruginosa*, *Staphylococcus aureus*, *Enterococcus faecium* and *Enterococcus faecalis*. Additionally, we estimated pooled worldwide resistance rates for these pathogens.

**Results:**

41 countries had the requisite data and were included in the study. Resistance and use rates were highly variable across countries, but *A. baumannii* resistance rates were uniformly higher, on average, than other organisms. High-income countries, particularly Sweden, Canada, Norway, Finland and Denmark, had the lowest DRIs; the countries with the highest DRIs, and therefore the lowest effectiveness of antibiotic therapy, were all low-income and middle-income countries.

**Conclusions:**

The DRI is a useful indicator of the problem of resistance. By combining data on antibiotic use with resistance, it captures a snapshot of how the antibiotics a country typically uses match their resistance profiles. This single measure of the effectiveness of antibiotic therapy provides a means of benchmarking against other countries and can, over time, indicate changes in drug effectiveness that can be easily communicated.

**Key questions**

**What is already known?**

- Antibiotic resistance is threatening global health, development and food security; however, measures for assessing and communicating the threat of resistance are lacking.
- The Drug Resistance Index (DRI) allows for global assessment of the relative efficacy of countries’ antibiotic therapy.

**What are the new findings?**

- Worldwide resistance rates for priority pathogens are variable across countries, but they are generally higher in low-income and middle-income countries (LMICs).
- The DRI scores demonstrate that not only do these countries suffer from a high burden of disease, but they also reflect a relatively lower level of antibiotic effectiveness due to less access to newer, more effective antibiotics.

**What do the new findings imply?**

- Resistance rates for the priority pathogens remain at a level that threatens public health, and the relative effectiveness of antibiotic therapy in LMICs is lower than in high-income countries.
- As surveillance for antibiotic resistance improves worldwide, the DRI can play a role in monitoring trends and the effectiveness of policies to curb overuse of antibiotics and increase access in LMICs.

## Introduction

Rising antibiotic resistance poses a significant threat to global health, development and food security. Resistance worldwide has been driven, in part, by antibiotic use, which has grown 66% since 2000.^1^ A large proportion of this consumption is unnecessary.^2–5^ Despite political commitments to address antimicrobial resistance made at the World Health Assembly and the 71st General Assembly of the United Nations,^6^ no effective system has been proposed to track progress at a global scale. A major hindrance to action is the complexity of evaluating and communicating the problem of antibiotic resistance, especially given the large set of combinations of antibiotics and bacteria. For example, methicillin-resistant *Staphylococcus aureus* (MRSA), which many in the public recognise as the first ‘superbug,’ is only one of several resistant pathogens that threaten human health.^7–9^ However, changes in the rates of MRSA may not be representative of the problem of resistance overall; even its name conveys only a subset of the problem—resistance to the beta-lactam class of antibiotics, to which methicillin belongs. MRSA is typically resistant to multiple classes of antibiotics, although the extent of resistance, and thus the potential threat to patient health, varies based on a pathogen’s genetic make-up and background. These nuances in resistance, and particularly the implications, can be difficult even for an audience of health professionals to grasp. Consequently, discussions regarding resistance have remained largely within the domain of scientific experts, and broader audiences have failed to understand the urgency of the antibiotic crisis.

Tracking trends in the effectiveness of antibiotic therapy across regions or countries poses an additional challenge. The need for, access to and use of antibiotics worldwide vary widely across countries and settings, as does the practice of antimicrobial stewardship.^10 11^ Factors that underlie these differences also evolve as economies grow and norms change. The combination of influences makes comparisons of performance among countries especially hard to measure.

To mitigate these measurement and communication problems, we developed a snapshot of the effectiveness of antibiotic therapy by country using the Drug Resistance Index (DRI)^12^ to demonstrate the ability to communicate the variations in antibiotic resistance and use. The DRI, which has been used in earlier studies for similar purposes,^13 14^ resembles composite price indices used in economics: it combines measurements of antibiotic consumption and resistance across multiple pathogen–organism combinations to create a single metric that represents an aggregate level of drug resistance. Similar to stock market indices that aggregate market valuations across companies of similar size or from specific sectors, different indices can be created at different geographical levels, from hospital to country, as well as for different types of bacterial infections (eg, skin and soft tissue infections or Gram-negative infections). Using the DRI to communicate gaps in the effectiveness of antibiotic therapy can contribute to global awareness of this growing problem and steer coordinated efforts to combat antimicrobial resistance. The data we present here demonstrate the wide disparities in relative effectiveness of antibiotic therapy globally and call attention to disparities in the problem of antibiotic resistance.

## Methods

The methodology for calculating the DRI has been described in previous publications.^12 15^ Briefly, we compute a composite index score for a unit of time by multiplying the proportion of each antibiotic used during the time period to treat a set of pathogens by the proportion of all isolates tested during the time period that were resistant to that drug. The resulting score is between 0 and 100, where 0 indicates 100% susceptibility and 100 indicates 100% resistance. The following equation is used to calculate the DRI for each country:

$DRI=\sum_{k}\rho_{k}^{t}q_{k}^{t}$(1)

where $\rho_{k}^{t}$ is the proportion of resistance among all included pathogens to drug *k* for time *t*, and $q_{k}^{t}$ is the proportion of drug *k* used for treatment of those pathogens in all drugs included in the index for time *t*. The time unit used for this study was a year.

We calculated country-level DRIs using a set of disease-causing organisms common to the countries in our analysis and considered by the WHO as priority pathogens^16^: *Acinetobacter baumannii*, *Escherichia coli*, *Klebsiella pneumoniae*, *Pseudomonas aeruginosa*, *Staphylococcus aureus*, *Enterococcus faecium* and *Enterococcus faecalis*. Antibiotic classes included aminoglycosides, broad-spectrum penicillins, carbapenems, cephalosporins, glycopeptides, narrow-spectrum penicillins and quinolones, although not all drugs were used to treat every pathogen. Table 1 lists the pathogen–antibiotic combinations included in the calculations.

**Table 1:** Pathogen–antibiotic combinations included in the Drug Resistance Index

*Acinetobacter baumannii*	Aminoglycosides. Cephalosporins (third generation). Fluoroquinolones. Carbapenems.
*Enterococcus faecalis*	Aminopenicillins. Aminoglycosides (high-level). Vancomycin.
*Enterococcus faecium*	Aminopenicillins. Aminoglycosides (high-level). Vancomycin.
*Escherichia coli*	Aminoglycosides. Aminopenicillins. Carbapenems. Cephalosporins (third generation). Fluoroquinolones.
*Klebsiella pneumoniae*	Aminoglycosides. Carbapenems. Cephalosporins (third generation). Fluoroquinolones.
*Pseudomonas aeruginosa*	Aminoglycosides. Carbapenems. Cephalosporins (third generation). Fluoroquinolones. Piperacillin-tazobactam.
*Staphylococcus aureus*	Oxacillin/Cefoxitin (methicillin-resistant *S. aureus*).

### Data

Data on resistance were obtained from ResistanceMap (www.resistancemap.org), a global repository of antibiotic resistance data for blood and cerebrospinal fluid (CSF) isolates from quality-assured and accredited hospitals and laboratory networks.^17^ The annual rates of resistance for each pathogen–antibiotic combination were obtained for all possible years. Antibiotic use data were obtained from IQVIA’s MIDAS database (IQVIA, Danbury, Connecticut, USA). IQVIA uses national sample surveys of antibiotic sales to develop estimates of the total volume of sales of each antibiotic molecule (or combination of molecules). Data from MIDAS were available in kilograms and converted to defined daily doses (DDDs)^1^ using the Anatomical Therapeutic Chemical Classification System (ATC/DDD, 2016) developed by the WHO Collaborating Centre for Drug Statistics Methodology.

To compare the DRI across countries, we used the most recent data available for each country. Countries were included if antibiotic use and resistance data were available for any year between 2012 and 2015. Because we were not able to obtain the full panel of pathogen–antibiotic combinations for all countries, we included a country if it had data for at least 5 of the 7 WHO priority pathogens, and 15 of the 25 possible pathogen–antibiotic combinations. To compare antibiotic use across countries, we calculated the DDDs per capita and measured the coefficient of variation (cov). Countries were analysed by income level using World Bank classifications for 2015.^18^ Comparison of resistance rates across countries is complicated by variations in the error of the sample estimates, due to differences in countries’ surveillance sample sizes and methodologies. To provide an improved estimate of worldwide resistance rates and to allow for pathogen–antibiotic resistance comparisons across countries, we used a meta-analysis framework to calculate pooled resistance rates for pathogen–antibiotic combination country comparisons.^19^ While meta-analysis methods are generally used to establish the effect of interventions, these methods can also be useful for establishing a more precise estimate of disease prevalence, and have been used for several diseases including prevalence of antibiotic resistance.^20 21^ We used the metafor package,^22^ a meta-analysis package for R,^23^ to calculate pooled resistance proportions using the Freeman-Tukey double arcsine transformation to control for prevalence values near the boundaries,^19^ and a random-effects model (DerSimonian-Laird method) due to heterogeneity in the prevalence rates. The variance of the proportions of resistant isolates was combined with a uniform SD based on IQVIA standardised methodology of the estimated DDD for each country to generate CIs for the DRI as the variance of the product of variables.^24 25^

## Results

**Figure 1:**
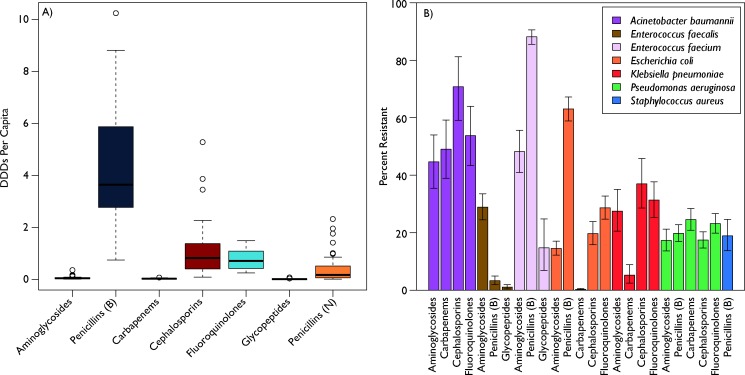
Global antibiotic use and resistance. (A) Global antibiotic use by antibiotic class. Each bar plots the variability in per capita antibiotic use measured in defined daily doses (DDDs). The black line is the median, the coloured bars are the quartiles, and the whiskers are the extremes. Additional outliers are plotted as circles. Penicillins were split into broad-spectrum, B, and narrow-spectrum, N. (B) Global antibiotic resistance rates for WHO priority pathogens. Each bar is the weighted average global resistance rate for the specified antibiotic–pathogen combination calculated using a meta-analysis framework, and the whiskers are the calculated 95% CIs. Resistance data come from ResistanceMap (resistancemap.cddep.org). Antibiotic use data come from the IQVIA MIDAS database. Source: IQVIA MIDAS, 2000–2015, IQVIA. All rights reserved.

A total of 41 countries had data on at least 15 pathogen–antibiotic combinations and 5 pathogens and were included in the study. Antibiotic use rates varied across countries (figure 1A); broad-spectrum penicillins were the most consumed class, but were relatively less variable (cov=53%) than narrow-spectrum penicillins (cov=135%) and cephalosporins (cov=97%). Despite the lower median use rates in low-income and middle-income countries (LMICs), the qualitative differences in variation were similar across LMICs and high-income countries (HICs) (online supplementary figure 1A,B). Resistance rates were also highly variable across countries (figure 1B). Overall *E. faecium* resistance to broad-spectrum penicillins was highest at 87% (95% CI 84 to 91), although *A. baumannii* resistance rates were, on average, the highest across all measured antibiotics. Resistance rates in LMICs were broadly higher than in HICs (online supplementary figure 1C,D), and variance was also generally higher in LMICs, except for *A. baumannii*, which had higher variance in resistance rates in HICs.

10.1136/bmjgh-2018-001315.supp1 Supplementary data

**Figure 2:**
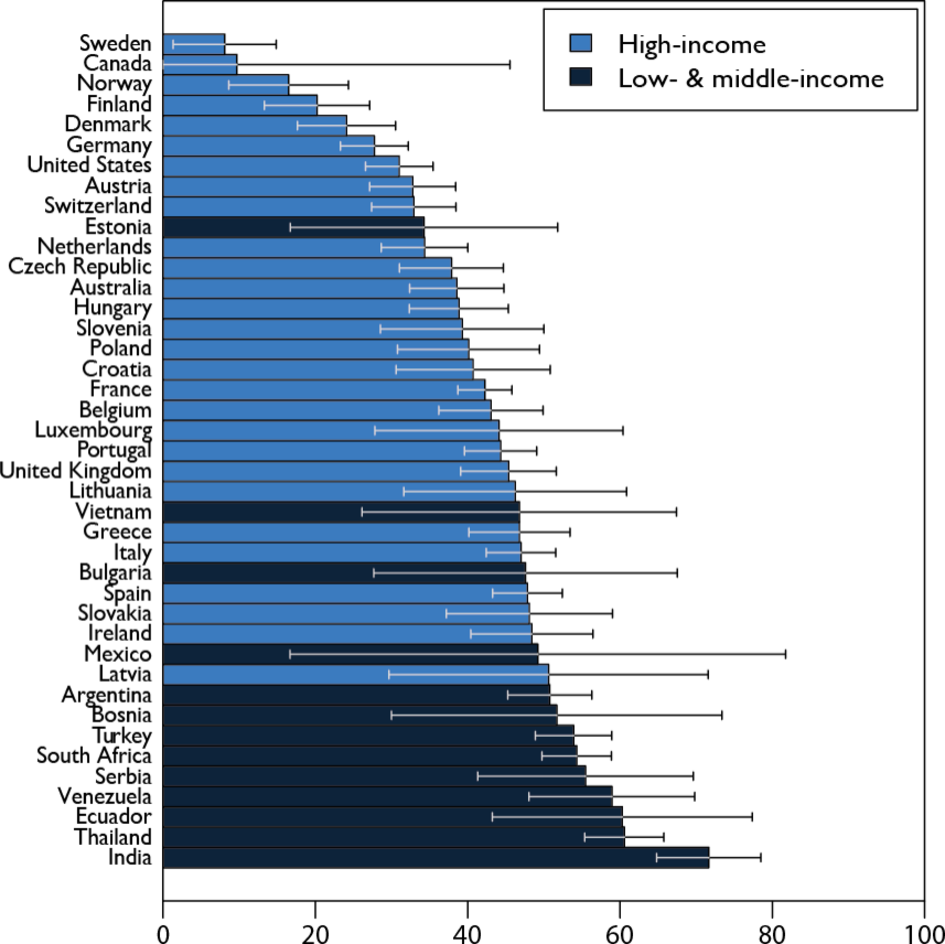
Drug Resistance Index (DRI) across countries. Each bar reports the DRI for countries reporting antibiotic resistance for 5 or more pathogens and for 15 or more pathogen–antibiotic combinations for at least 1 year between 2012 and 2015. Data for the most recent year are shown. All countries included had resistance data for all seven antibiotic classes except Vietnam, which did not have resistance data for glycopeptides. Country income classifications were based on World Bank analytical classifications for fiscal year 2015.

Reflecting the variance in resistance rates, we found that HICs had generally lower DRI rates than LMICs (figure 2). The five countries with the lowest DRIs were Sweden, Canada, Norway, Finland and Denmark, all HICs, four of which are in Northern Europe. In contrast, those with the highest DRIs, and therefore the lowest relative effectiveness of antibiotic therapy, were predominantly LMICs, including India, Thailand, Ecuador and Venezuela. These findings were consistent even after restricting the analysis to countries that had all 25 pathogen–antibiotic combinations.

**Figure 3:**
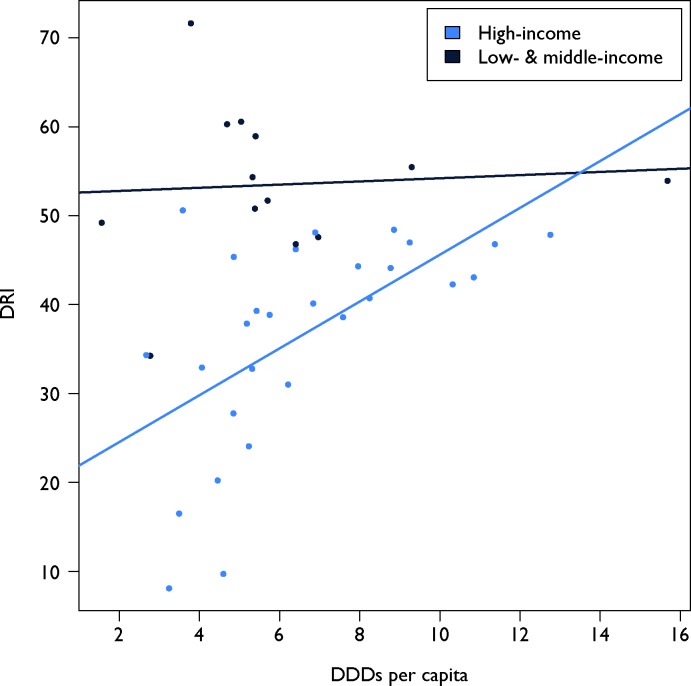
Drug Resistance Index (DRI) for each country compared with its per capita antibiotic use in defined daily doses (DDDs) per 1000 individuals. The light-blue dots are individual high-income countries and the dark-blue dots are individual low-income and middle-income countries. The lines, which are coloured similar to the dots, are the linear trend of the corresponding colour dots. DRI and DDD data are the most recent available for each country between 2012 and 2015. Country income classifications were based on World Bank analytical classifications for fiscal year 2015. Antibiotic use data come from the IQVIA MIDAS database. Source: IQVIA MIDAS, 2000–2015, IQVIA. All rights reserved.

**Figure 4:**
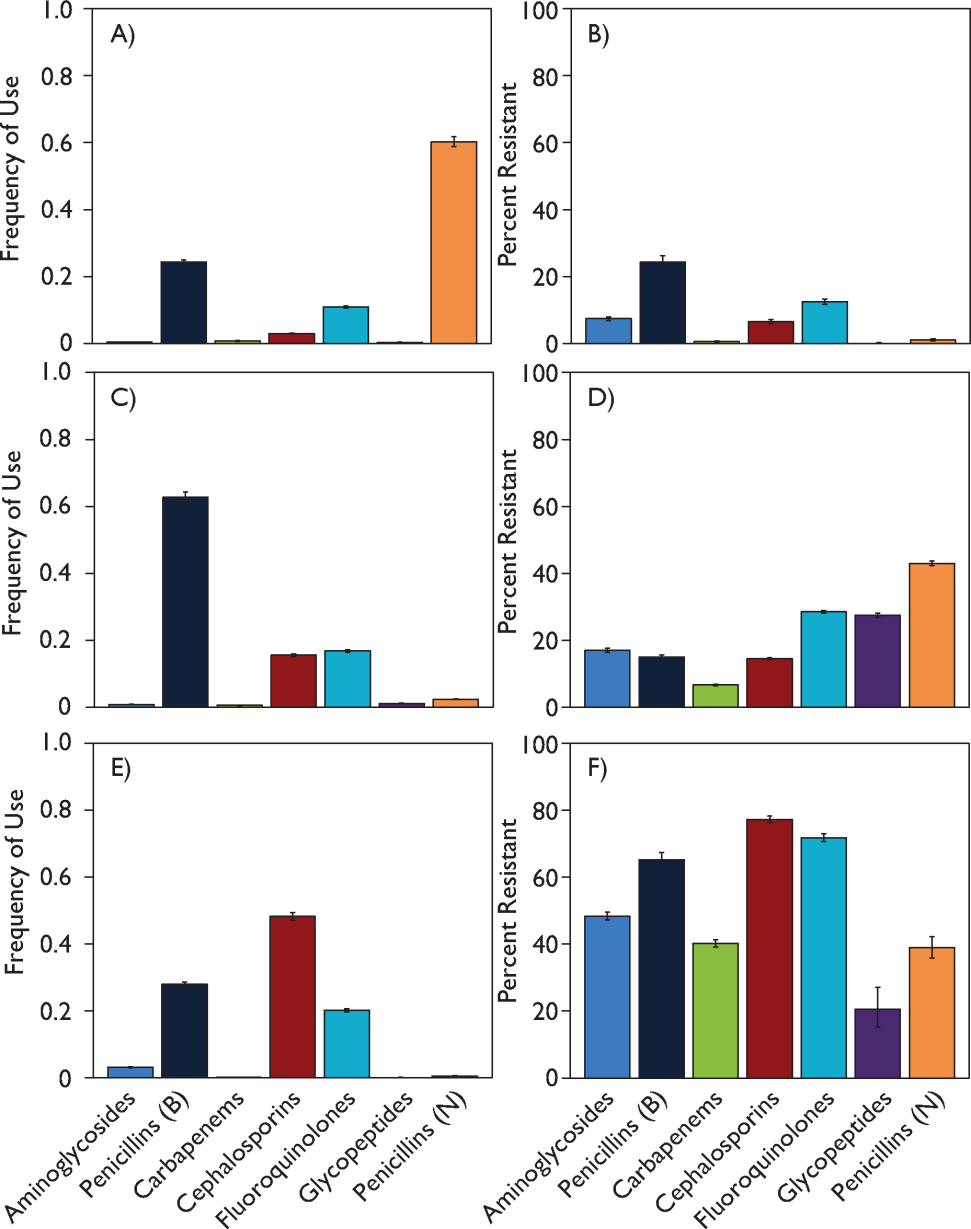
Underlying DRI data for Sweden, USA and India, by antibiotic class, 2015. The top row (A, B) reports the antibiotic use and antibiotic resistance for Sweden in 2015; the middle row (C, D) and the bottom row (E, F) show the corresponding information for USA and India, respectively. Antibiotic use graphs are the proportion of each drug consumed of the total antibiotic use included. Resistance reflects the percentage resistant rates reported from ResistanceMap (resistancemap.cddep.org). Antibiotic use data come from the IQVIA MIDAS database. Source: IQVIA MIDAS, 2000–2015, IQVIA. All rights reserved.

Both use and resistance rates affect the derivation of the DRI for a country. On the use side, the relationship between a country’s DRI and the frequency of antibiotic use (DDD) per capita varied widely among countries (figure 3). This relationship between total use and the DRI also differed between HICs and LMICs. Although a higher per capita DDD was associated with a higher DRI in HICs, this relationship was not as strong in LMICs. Thus, despite having the highest DRIs, India, Ecuador, Thailand and Venezuela had relatively lower antibiotic use per person. However, the DRI reflects use related to resistance; in that sense, the relatively higher resistance rates in LMICs, particularly for broad-spectrum penicillins, weighs heavily in the resulting DRI. For instance, data for Sweden and India (the countries with the lowest and highest DRIs, respectively) as well as the USA (which is in the middle) showed markedly different use and resistance patterns (figure 4). In Sweden, narrow-spectrum (0.60) and broad-spectrum (0.24) penicillins, followed by fluoroquinolones (0.11), composed the majority of antibiotics consumed in 2015, whereas in India cephalosporins (0.48), broad-spectrum penicillins (0.28) and fluoroquinolones (0.20) predominated. In the USA, broad-spectrum penicillins (0.63) captured the highest frequency of use, followed by fluoroquinolones (0.17) and cephalosporins (0.16). Resistance to broad-spectrum penicillins, the class of antibiotics with the highest rate of resistance in all the countries, is thus a more important component of the DRI for the USA and India relative to Sweden because Sweden has lower relative use of these drugs. Similarly, the high rate of resistance to cephalosporins in India has a comparatively greater effect because of the country’s higher relative use of cephalosporins. Underlying analyses of other LMICs showed resistance patterns similar to India’s, and HICs with low DRIs demonstrated levels of antibiotic use and resistance comparable with Sweden’s. However, HICs with low to moderate DRIs, such as Australia, Poland and France, tended to have increased resistance to and decreased consumption of narrow-spectrum penicillins. Not all HICs had low DRIs: Spain, Greece, Italy and Ireland had high values, reflecting their higher rates of both resistance and use.

## Discussion

Formulating a response to the global challenge posed by rising antibiotic resistance requires communicating the problem in a clear manner. However, the problem of resistance is complicated because infections can be caused by many different pathogens, and each infection can be treated by a multitude of drugs. To date, this information has largely been communicated through either detailed pathogen susceptibility breakdowns targeted specifically at clinicians, or stories of ‘superbugs’ relayed by the popular press. The DRI^12^ attempts to bridge this gap by reducing the complexity of communicating the resistance problem by combining drug use and resistance across pathogens into a single measure. This single value makes communicating the relative effectiveness of antibiotic therapy significantly easier. Here we applied this approach to generate DRI values for countries around the world.

Similar to stock market indices, the DRI provides a useful indicator that allows comparisons across countries. By combining antibiotic use and resistance, it shows how a country’s use of antibiotic therapy relates to levels of drug resistance. Thus, a country with high per capita drug use would not have a high DRI if resistance rates to the most frequently used drugs were low. Conversely, high rates of resistance would lead to a high DRI even if per capita antibiotic use were relatively low. In our analyses, we found a large spread in the DRI, yet factors driving variation in the DRI differed by a country’s income level. In HICs, variation was largely related to antibiotic use. Higher DDDs per capita were associated with higher DRIs in large part because increased antibiotic use leads to increases in resistance. The underlying mix of antibiotics used likely also played a role. For instance, although resistance patterns in France were generally similar to those in Denmark, the two countries had drastically different patterns of antibiotic consumption—most notably, Denmark’s relatively low use of broad-spectrum penicillins and relatively high use of narrow-spectrum penicillins. Because resistance to these classes of drugs was fairly similar in both countries, it was the differences in use that drove the spread between the countries’ DRIs. Different factors led to variation in the DRIs among LMICs, since these countries’ higher per capita DDDs were associated with lower DRIs. Underlying data showed that for nearly all pathogen–organism combinations in LMICs, higher rates of resistance relative to HICs led to higher DRIs despite lower per capita antibiotic use. The reversed relationship in LMICs may reflect a number of factors: (1) systemic differences in healthcare systems that increase transmission of resistant pathogens; (2) disparities in measurement (eg, surveillance biased towards finding resistance); and (3) different subnational patterns of antibiotic use (eg, if use were concentrated in a small subset of the population, greater per capita use would lead to higher resistance).

The DRI provides a clear way of evaluating and comparing the problem of resistance across countries. By encapsulating the multiple relationships between pathogen susceptibility and antibiotic use into an easily understood metric that is comparable across time and space, the DRI reveals where resistance poses a greater problem and helps communicate the issues of resistance to lay audiences. Although we calculated a cross-sectional DRI in this study, trends in the DRI can be helpful in determining the effectiveness of interventions and could provide early warning of future issues. Unlike stock indices, the DRI is tied to fundamental changes in antibiotic use and resistance. The DRI also captures affordability constraints as countries move to newer, more expensive antibiotics: one reason we observed higher DRIs in LMICs is that these countries have not yet made this switch. High-income and low-income countries with similar resistance profiles could have DRIs that are quite different, reflecting the affordability of newer antibiotics in the HIC (as measured by use rates). However, the ultimate utility of the DRI depends on the quality of surveillance, which provides the underlying data on antibiotic use and resistance. Lab capacity for surveillance is limited in many LMICs, particularly those in sub-Saharan Africa. Even in HICs, surveillance mainly consists of passive data collection used for patient treatment, and as a result broader community trends in resistance are by and large absent from discussions of antimicrobial resistance. Increasing lab capacity in LMICs would improve patient therapy and allow for richer, more detailed surveillance, although this is no easy task. Setting up a lab is complex; however, it is often more difficult once established to maintain lab staff, ensure funds for ongoing surveillance, and most importantly get clinician buy-in to provide isolates for patient care that can then be used for surveillance. Long-term government funding and commitments are necessary to build this capacity.

Our study has several limitations. First, data on both antibiotic resistance and use were available for only 11 LMICs, all of them either lower income or upper-middle-income countries. Because of a lack of surveillance both for use and resistance in low-income countries, the problem of resistance in these countries is not well understood. Second, the quality of data for both use and resistance may not be uniform across HICs and LMICs. Although we had no way of assessing quality differences, we did generate CIs based on inverse-variance weighting of the resistance data and standardised variance in the use data. Third, antibiotic use data come from a single source of harmonised data on global antibiotic consumption. Because there is no alternative repository of data, we were unable to determine whether systematic biases exist in this data source for LMICs, although for HICs the data are highly correlated with data from Europe.^1^ Fourth, we only used data on resistance for blood and CSF isolates, which are the only type collected by ResistanceMap, to ensure the resistance data are for infections and not contaminants. Without data on other types of isolates that cover other pathogen–antibiotic combinations, the DRIs presented here may not be fully representative of the effectiveness of antibiotic therapy in a country. However, the DRI can be calculated in different ways, and as additional data become available new indexes can be calculated, similar to how major stock indexes have proliferated over the years. Lastly, we focused on the most common disease-causing organisms globally; however, other country-specific pathogens that contribute substantially to a country’s burden of drug resistance may be important for understanding the efficacy of antibiotic therapy at a country level even if not comparable across countries. Future research should investigate detailed country-specific data and use different drug–pathogen combinations to capture within-country dynamics of antibiotic use and resistance while still making comparisons across countries.

The introduction of the DRI^12^ was a first step in evaluating the relative problems of resistance and communicating the findings to a broad audience. Here we used the DRI to demonstrate how the problems of resistance vary widely by geography, reflect underlying trends in antibiotic use and infer potential factors driving the differences among countries. With improvements in surveillance, the DRI could be used to monitor trends in resistance and the effectiveness of the global response. Detailed study of the factors driving DRI differences can also provide insight into potential solutions to the problem. However, fundamentally, the DRI is a relatively easy mechanism for communicating the problem of resistance, and as such can help drive interest and track progress. Future applications of the DRI could include hospital-level comparisons at regional and national levels that could be more readily understood as an indicator of hospital quality.
