# Supplementary material for: Tracking global trends in the effectiveness of antibiotic therapy using the Drug Resistance Index
Source: BMJ Glob Health. 2019 Apr 11;4(2):e001315. doi: 10.1136/bmjgh-2018-001315 (PMC6509601; doi:10.1136/bmjgh-2018-001315)
Supplement: Supplementary data [file bmjgh-2018-001315supp001.pdf]

Supplementary Material

for

Tracking Global Trends in the Effectiveness of Antibiotic Therapy Using the Drug Resistance Index

Eili Y. Klein PhD,[1,2,3] Katie K. Tseng MPH,[1] Suraj Pant BA,[1] Ramanan Laxminarayan PhD[1,4,5]

1 Center for Disease Dynamics, Economics & Policy, Washington, DC 20005
2 Department of Emergency Medicine, Johns Hopkins School of Medicine, Baltimore, MD 21209
3 Department of Epidemiology, Johns Hopkins Bloomberg School of Public Health, Baltimore, MD 21205
4 Princeton Environmental Institute, Princeton University, Princeton, NJ 08544
5 Department of Global Health, University of Washington, Seattle, WA 98104

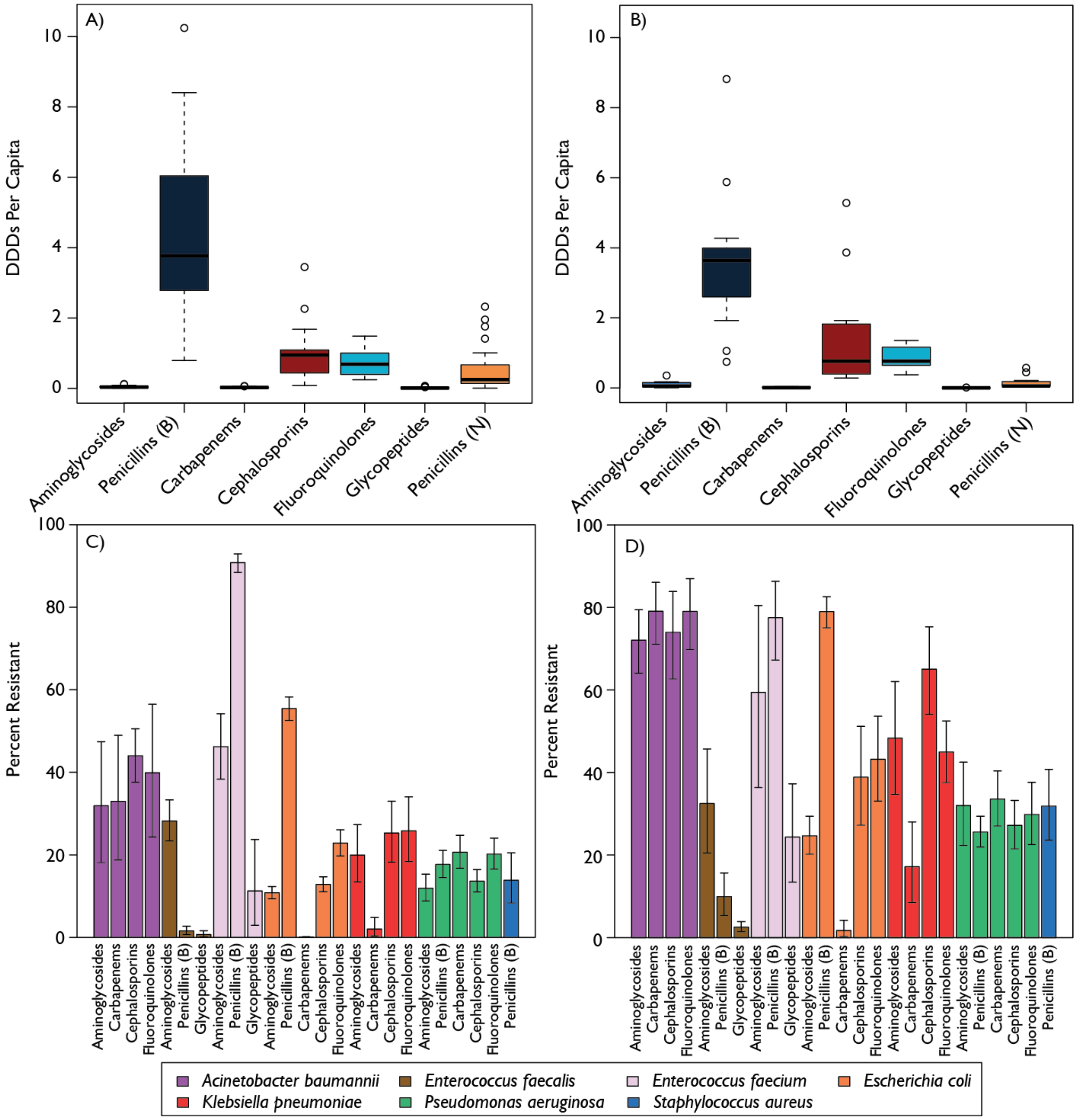

Supplementary Figure 1: Global antibiotic use and resistance, by income class. (A,B) Each bar plots the variability in per capita antibiotic use measured in defined daily doses (DDDs) for high-income countries (A) and low- and middle-income countries (B). The black line is the median, the colored bars are the quartiles, and the whiskers are the extremes. Additional outliers are plotted as circles. Penicillins were split into broad-spectrum, B, and narrow-spectrum, N. (C,D) Each bar is the weighted average global resistance rate for the specified antibiotic-pathogen combination in high-income countries (C) and low- and middle-income countries (D) calculated using a meta-analysis framework. Resistance data come from ResistanceMap (resistancemap.cddep.org). Antibiotic use data come from the IQVIA MIDAS database. Source: IQVIA MIDAS, 2000–2015, IQVIA Inc. All rights reserved.

Supplementary Table 1: Global Antibiotic Use Rates by Antibiotic Class

	Median DDDs per capita (IQR)		
**Antibiotic Class**	**Global**	**High Income Countries**	**Low- and Middle-Income Countries**
Aminoglycosides	41 (23-62)	35 (22-55)	59 (33-155)
Broad-spectrum penicillins	3,644 (2,775-5,864)	3,767 (2,783-6,040)	3,642 (2,603-3,990)
Carbapenems	23 (10-33)	24 (13-34)	10 (3-26)
Cephalosporins	824 (408-1,368)	941 (441-1,091)	762 (397-1,824)
Fluoroquinolones	709 (422-1,082)	688 (398-1,002)	762 (648-1,162)
Glycopeptides	8 (4-15)	11 (7-17)	4 (1-6)
Narrow-spectrum penicillins	170 (59-495)	248 (142-666)	58 (49-179)

Supplementary Table 2: Global Antibiotic Resistance Rates by Organism and Antibiotic Class

		Percent Resistant (Confidence Interval)		
**Organism**	**Antibiotic Class**	**Global**	**High Income Countries**	**Low- and Middle-Income Countries**
Acinetobacter baumannii	Aminoglycosides	44.7 (35.4-54.0)	31.9 (18.1-47.4)	72.1 (64.0-79.4)
Acinetobacter baumannii	Carbapenems	49.0 (38.9-59.2)	33.0 (18.7-49.0)	79.1 (71.1-86.1)
Acinetobacter baumannii	Cephalosporins	70.8 (59.1-81.2)	44.0 (37.6-50.5)	74.0 (62.7-83.9)
Acinetobacter baumannii	Fluoroquinolones	53.7 (43.3-64.0)	39.9 (24.4-56.5)	79.0 (69.8-87.0)
Enterococcus faecalis	Aminoglycosides	28.9 (24.5-33.6)	28.2 (23.3-33.3)	32.5 (20.5-45.7)
Enterococcus faecalis	Broad-spectrum penicillins	3.3 (1.9-4.9)	1.5 (0.7-2.7)	9.9 (5.3-15.7)
Enterococcus faecalis	Glycopeptides	1.1 (0.5-1.9)	0.7 (0.1-1.6)	2.5 (1.4-3.9)
Enterococcus faecium	Aminoglycosides	48.2 (40.9-55.6)	46.2 (38.3-54.2)	59.4 (36.4-80.5)
Enterococcus faecium	Broad-spectrum penicillins	88.2 (85.5-90.6)	90.8 (88.5-92.9)	77.5 (67.2-86.3)
Enterococcus faecium	Glycopeptides	14.7 (6.9-24.8)	11.3 (2.9-23.7)	24.3 (13.4-37.2)
Escherichia coli	Aminoglycosides	14.5 (12.2-17.0)	10.8 (9.4-12.3)	24.7 (20.2-29.4)
Escherichia coli	Broad-spectrum penicillins	63.1 (58.9-67.2)	55.4 (52.6-58.3)	79.0 (75.1-82.6)
Escherichia coli	Carbapenems	0.3 (0.1-0.6)	0.1 (0.0-0.2)	1.7 (0.3-4.2)
Escherichia coli	Cephalosporins	19.7 (15.9-23.8)	12.8 (11.1-14.7)	38.9 (27.2-51.2)
Escherichia coli	Fluoroquinolones	28.6 (24.7-32.8)	22.8 (19.7-26.1)	43.2 (33.1-53.6)
Klebsiella pneumoniae	Aminoglycosides	27.5 (20.5-35.1)	19.9 (13.5-27.3)	48.3 (34.7-62.0)
Klebsiella pneumoniae	Carbapenems	5.2 (2.5-8.9)	2.0 (0.4-4.8)	17.2 (8.5-28.0)
Klebsiella pneumoniae	Cephalosporins	37.0 (28.6-45.8)	25.3 (18.3-33.0)	65.1 (54.1-75.3)
Klebsiella pneumoniae	Fluoroquinolones	31.4 (25.3-37.7)	25.8 (18.4-34.0)	45.0 (37.6-52.5)
Pseudomonas aeruginosa	Aminoglycosides	17.3 (13.7-21.2)	11.9 (8.8-15.3)	32.0 (22.3-42.5)
Pseudomonas aeruginosa	Broad-spectrum penicillins	19.8 (16.9-22.8)	17.6 (14.5-21.1)	25.6 (21.9-29.4)
Pseudomonas aeruginosa	Carbapenems	24.5 (20.8-28.5)	20.6 (16.8-24.8)	33.6 (27.0-40.4)
Pseudomonas aeruginosa	Cephalosporins	17.4 (14.7-20.3)	13.6 (11.0-16.4)	27.2 (21.5-33.2)
Pseudomonas aeruginosa	Fluoroquinolones	23.2 (19.8-26.7)	20.2 (16.6-24.0)	29.8 (22.6-37.6)
Staphylococcus aureus	Narrow-spectrum penicillins	18.9 (13.8-24.6)	13.8 (8.3-20.5)	31.9 (23.6-40.8)

Supplementary Table 3: Drug Resistance Index

Country	DRI (Confidence Interval)
Sweden	8.1 (1.3-14.9)
Canada	9.7 (0.0-45.5)
Norway	16.5 (8.6-24.3)
United States	18.0 (15.0-20.9)
Finland	20.2 (13.3-27.1)
Denmark	24.1 (17.6-30.5)
Germany	27.7 (23.3-32.2)
Austria	32.8 (27.1-38.4)
Switzerland	32.9 (27.4-38.5)
Estonia	34.2 (16.7-51.8)
Netherlands	34.3 (28.6-40.0)
Czech Republic	37.9 (31.1-44.7)
Australia	38.6 (32.4-44.7)
Hungary	38.8 (32.3-45.3)
Slovenia	39.3 (28.5-50.0)
Poland	40.1 (30.8-49.4)
Croatia	40.7 (30.6-50.8)
France	42.3 (38.7-45.8)
Belgium	43.1 (36.2-49.9)
Luxembourg	44.1 (27.8-60.4)
Portugal	44.3 (39.6-49.0)
United Kingdom	45.4 (39.1-51.6)
Lithuania	46.2 (31.6-60.9)
Vietnam	46.8 (26.1-67.4)
Greece	46.8 (40.2-53.4)
Italy	47.0 (42.4-51.6)
Bulgaria	47.6 (27.7-67.5)
Spain	47.8 (43.3-52.4)
Slovakia	48.1 (37.2-59.0)
Ireland	48.4 (40.4-56.4)
Mexico	49.2 (16.7-81.7)
Latvia	50.6 (29.7-71.6)
Argentina	50.8 (45.3-56.3)
Bosnia and Herzegovina	51.7 (30.0-73.4)
Turkey	53.9 (48.9-58.9)
South Africa	54.3 (49.8-58.9)
Serbia	55.5 (41.3-69.6)
Venezuela	58.9 (48.1-69.8)
Ecuador	60.3 (43.2-77.4)
Thailand	60.6 (55.4-65.8)
India	71.6 (64.8-78.5)
